# Supplementary material for: HIV, asymptomatic STI, and the rectal mucosal immune environment among young men who have sex with men
Source: PLoS Pathog. 2023 May 30;19(5):e1011219. doi: 10.1371/journal.ppat.1011219 (PMC10256205; doi:10.1371/journal.ppat.1011219)
Supplement: S1 Table — (DOCX) [file ppat.1011219.s001.docx]

| **Cell subsets characterized in blood** | **Cell subsets characterized in RM** |
| --- | --- |
| CD4+ T cells | CD4+ T cells |
| CD4+_memory(mem)_ T cells | CD4+_memory(mem)_ T cells |
| CD4+_mem_ CCR5+ T cells | CD4+_mem_ CCR5+ T cells |
| CD4+_mem_ Ki67+ T cells | CD4+_mem_ Ki67+ T cells |
| CD4+_mem_ α4β7+ T cells | CD4+_mem_ α4β7+ T cells |
| Treg cells (CD25+CD25HiFOXP3) | Treg cells (CD25+CD25HiFOXP3 |
| CD8+ T cells | CD8+ T cells |
| CD8+_mem_ T cells | CD8+_mem_ T cells |
| CD8+_mem_ Ki67+ T cells | CD8+_mem_ Ki67+ T cells |
| CD4+ IL–17+ T cells | CD4+ IL–17+ T cells |
| CD4+ IFNγ+ T cells | CD4+ IFNγ+ T cells |
| CD4+ TNFα+ T cells | CD4+ TNFα+ T cells |
| CD8+ IFNγ+ T cells | CD8+ IFNγ+ T cells |
| CD8+ TNF+ T cells | CD8+ TNF+ T cells |
| MAIT cells (CD3+CD161+TCR Vα7.2+) | MAIT cells (CD3+CD161+TCR Vα7.2+) |
| γδ T cells (CD3+CD161-TCR γδ+) | γδ T cells (CD3+CD161-TCR γδ+) |
| NK CD16– CD56+ cells | NK CD16– CD56+ cells |
| NK CD16+ CD56_dim_ cells | NK CD16+ CD56_dim_ cells |
| CD1c+ mDC cells (CD20-CD3-CD1c+CD16-) | CD1c+ mDC cells (CD20-CD3-CD1c+CD16-) |
| pDC cells (CD20-CD3-CD123+CD11c-) | pDC cells (CD20-CD3-CD123+CD11c-) |
| B cells (HLADR+ CD20+ CD3–) | B cells (HLADR+ CD20+ CD3–) |
| Monocytes | Macrophages (CD20-CD3-CD163+CD16-) |
| Inflammatory monocytes | Neutrophils (CD45+CD16+CD66b+) |
|  | CD4+ T_resident memory(rm)_ (CD4+CD69+) |
|  | CD8+ T_rm_ (CD8+CD69+CD103+) |
|  | CD4+ and CD8+ nonT_rm_ (CD69-CD103-) |
