# Supplementary figures and images for: HIV, asymptomatic STI, and the rectal mucosal immune environment among young men who have sex with men
Source: PLoS Pathog. 2023 May 30;19(5):e1011219. doi: 10.1371/journal.ppat.1011219 (PMC10256205; doi:10.1371/journal.ppat.1011219)

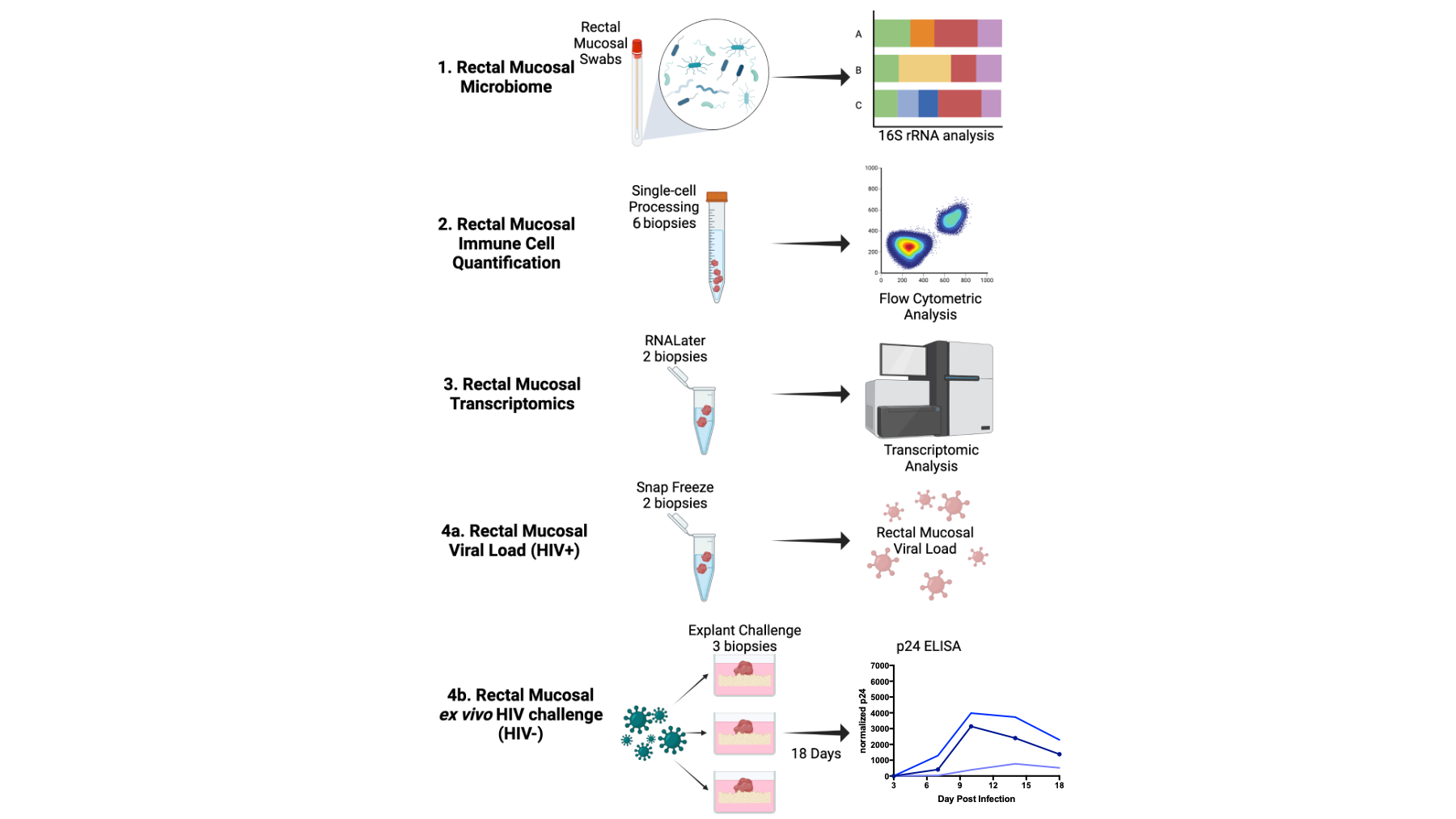

Supplement: S1 Fig — Each study participant underwent rigid sigmoidoscopy for the collection of study specimens. Rectal mucosal swabs were collected for 16S rRNA microbiome sequencing. Six rectal mucosal pinch biopsies were collected for immune cell quantification via flow cytometry. Two rectal mucosal pinch biopsies were collected for RNAseq transcriptomic analysis. For YMSM with HIV, two rectal mucosal pinch biopsies were collected for HIV viral load detection. For YMSM without HIV, three rectal mucosal pinch biopsies were collected for our ex vivo HIV challenge. Figure created with BioRender.com. (TIFF) [file ppat.1011219.s002.tiff]

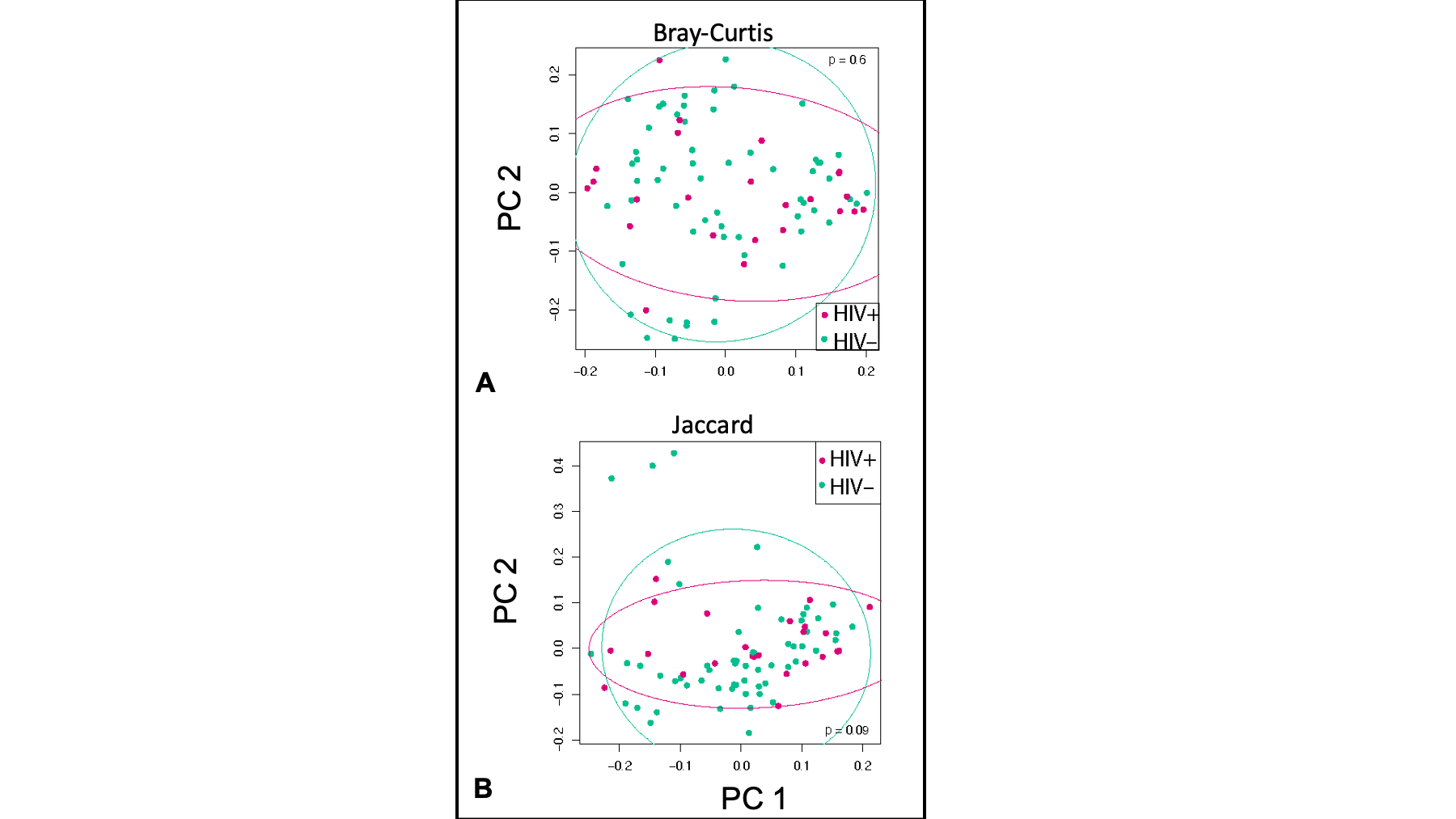

Supplement: S2 Fig — (A) Bray-Curtis and (B) Jaccard measures of beta diversity both demonstrated no significant differences between the microbiome composition of YMSM with and without HIV. Pink dots represent HIV-positive YMSM; teal dots represent HIV-negative YMSM. (TIFF) [file ppat.1011219.s003.tiff]

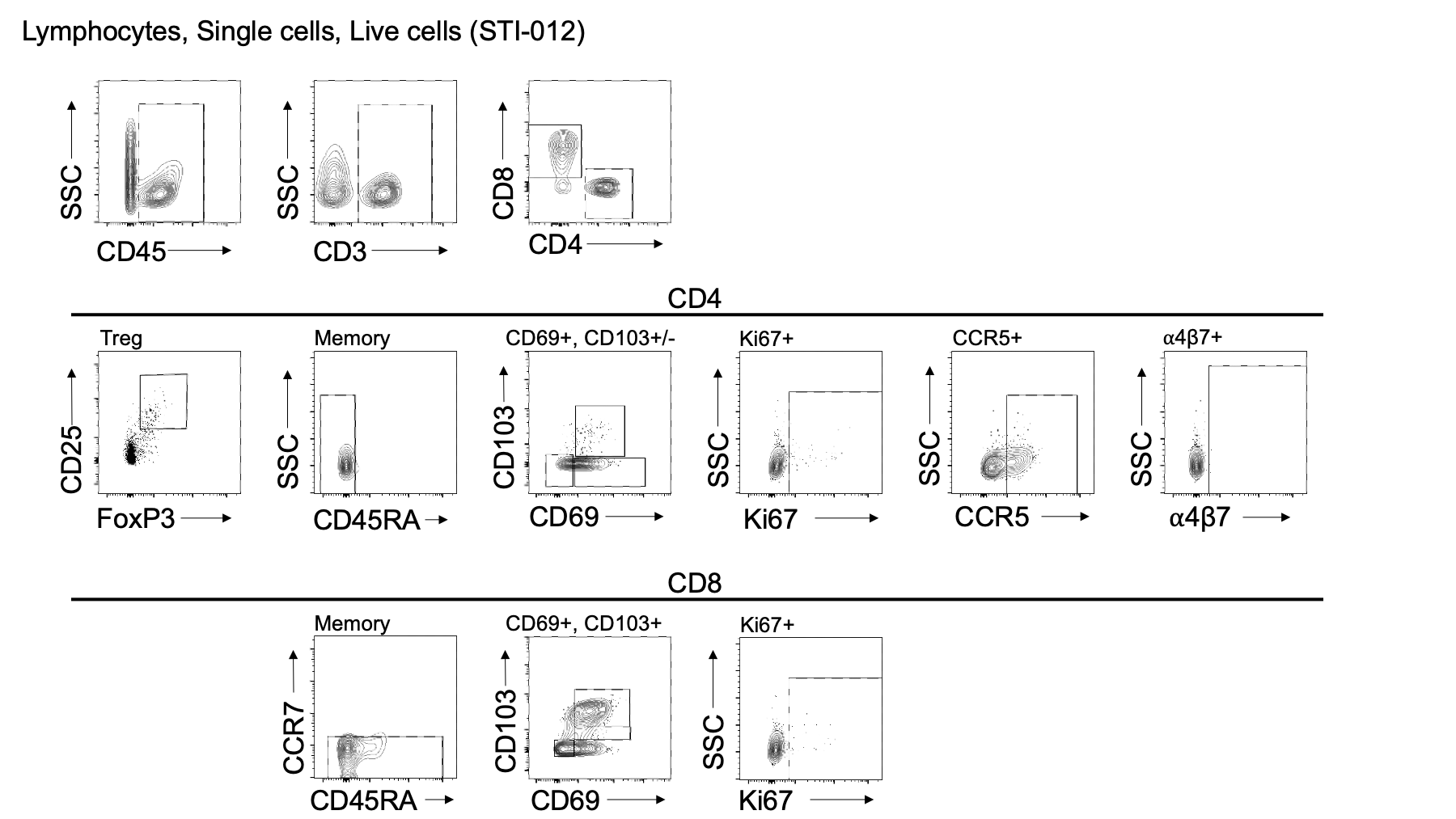

Supplement: S3 Fig — Lymphocytes were identified by forward and side scatter. CD45+ cells were then isolated, followed by CD3+ cells which were separated into CD4+ and CD8+ subsets. Memory CD4 cells were identified by excluding CD45RA+ cells. CD69 marker was then used to divide CD4+ cells into tissue resident and non-tissue resident populations. Memory CD8 + cells were designated as being CCR7- and CD45RA+/-. Among memory CD8+ T cells, both CD69 and CD103 markers were used to designate tissue resident populations. Memory CD4+ populations, including tissue resident and non-tissue resident subsets, were then assessed for expression of CCR5, α4β7, and Ki67. (TIFF) [file ppat.1011219.s004.tiff]

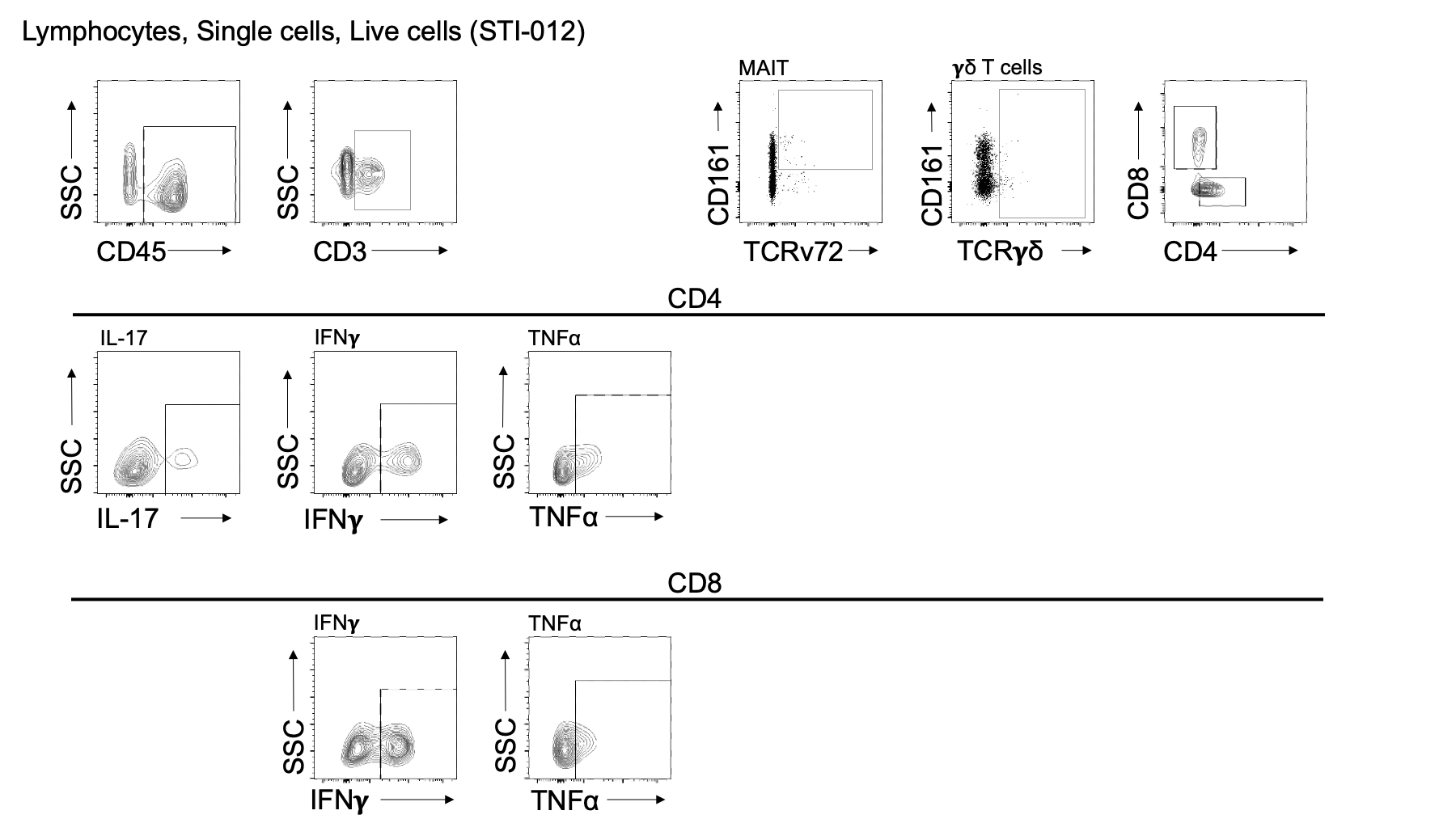

Supplement: S4 Fig — Rectal CD4+ and CD8+ MMCs were stimulated for 4 hours with PMA/Ionomycin and stained for indicated cytokines. Live cells were identified by live/dead staining and lymphocytes were identified by forward and side scatter. CD45+ cells were then isolated, followed by CD3+ cells which were separated into CD4+ and CD8+ subsets. Stimulated CD4+ T cells were assessed for IL-17A, IFNγ, and TNFα cytokine production and stimulated CD8+ T cells for IFNγ and TNFα. Abbreviations: MMC, mucosal mononuclear cells, PMA, phorbol myristate acetate, IFNγ, interferon gamma, TNFα, tumor necrosis factor alpha. (TIFF) [file ppat.1011219.s005.tiff]

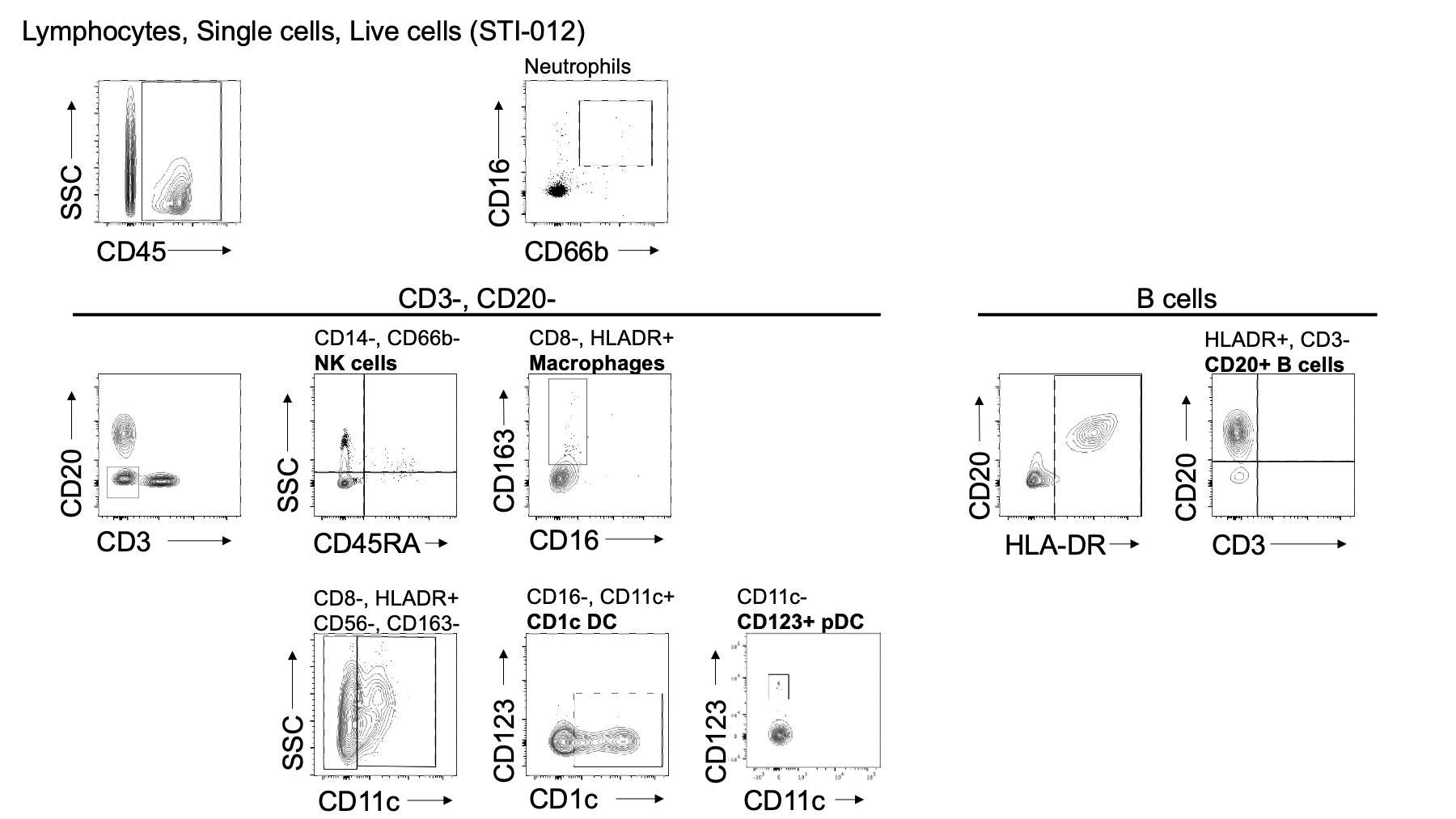

Supplement: S5 Fig — Representative flow panel to identify non-T-cell immune cell subsets: Lymphocytes were identified via forward and side scatter, singlets, and live cells. Neutrophils were CD66+ CD45+ cells. CD45+CD3-CD20- cells were further divided by negative gating to CD56+ and CD56+CD16+ NK, macrophages by CD16, CD163, and HLA-DR expression, while CD1c+ and pDC were identified with CD11c, CD1c, and CD123. B-cells were defined by HLA-DR and CD20 expression. (TIFF) [file ppat.1011219.s006.tiff]
