# Supplementary material for: HIV, asymptomatic STI, and the rectal mucosal immune environment among young men who have sex with men
Source: PLoS Pathog. 2023 May 30;19(5):e1011219. doi: 10.1371/journal.ppat.1011219 (PMC10256205; doi:10.1371/journal.ppat.1011219)
Supplement: Attachment — Submitted filename: PLOS Pathogens Cover Letter.docx [file ppat.1011219.s007.docx]

Dear PLOS Pathogens Editorial Staff,

Thank you for the opportunity to submit a revised draft of our manuscript titled “HIV, asymptomatic STI, and the rectal mucosal immune environment among young men who have sex with men” to *PLOS Pathogens*. We appreciate the time and effort that you and the reviewers have dedicated to providing your valuable feedback on this manuscript and are grateful for your insightful comments.

Below please find a point-by-point response to the reviewers’ comments and concerns.

**Part II – Major Issues: Key Experiments Required for Acceptance**

**Reviewer #1:** (No Response)

**Reviewer #2:** none

**Reviewer #3:**

**Question 1.** What was the median and range of time since HIV diagnosis for these individuals? And time on ART? While confounding effects from MSM status have caused inconsistent results across studies, several studies have been performed that matched HIVpos and HIVneg for MSM status. Some of these studies show no significant differences in the gut microbiome of HIVpos individuals when looking early in infection (10.1016/j.ebiom.2022.104286), but studies of MSM-matched chronically-infected adults show significant differences compared to HIVneg (DOI:10.1038/s41598-018-32585-x, 10.1038/s41467-020-16222-8, DOI:10.1038/s41598-017-06675-1). It will be helpful for readers to know if these HIVpos are early in their infection and whether that may be why no differences in the microbiome are seen.

**Response 1:** Unfortunately, data on time since HIV diagnosis are not available for our study cohort. Time since HIV diagnosis is notoriously difficult to obtain and can be unreliable, even in well characterized clinical cohorts, as people often migrate between clinics with disparate medical records. Of note, our cohort was fairly homogenous: young MSM (median age 21-22 years) with >80% identifying as Black men, all enrolled in Atlanta, Georgia, and all taking anti-retroviral therapy. To our knowledge, none of our participants acquired HIV at birth and all could be considered to have chronic HIV infection. Therefore, one might assume time from HIV infection to be relatively recent (<5-7 years), but not acute/early, given available data on sexual debut among MSM. Given the young age of participants, one might also assume that participants were only exposed to modern ART regimens for HIV treatment. Given the demographic and behavioral similarities of participants in our cohort and the relatively high median CD4 count (>600 cells/uL) of participants with HIV, it may be expected to see no differences in the composition of the rectal mucosal microbiota between young MSM with and without HIV based on the disparate literature available to date on this topic.

We have added a more thorough discussion of the above to the microbiome subsection of the Discussion (bottom of pg. 18) as the reviewer suggests.

**Question 2.** An aspect of the analytical design makes it difficult to confirm the authors’ conclusion that HIV has a greater impact on immune cell subsets than STIs. When HIVpos are compared to HIVneg, the difference between them is one infectious agent that causes its own unique skewing of immune subset proportions. When comparing STIpos to STIneg, several infections are grouped which may have different effects on immune cell subsets, making groupwise comparisons problematic. Groupwise comparisons test whether a group as a whole differs in a common, concerted way from another group. If there are multiple sub-groups that are inherently different from each other, this contributes noise that would hamper a statistical test’s assessment of significant differences. This would also interfere with systems-level analyses like in 2A. If three sub-groups were present within the STIpos that each had their own unique signature of immune cell subsets, they may be equally different from the STIneg and from each other, in which case there would be no statistically significant difference between STIpos and STIneg. Indeed, the STIpos group appears to have more intra-group variance in Figure 2A, which one might expect if there are unique influences on immune cells from the different STI types (Neisseria, Chlamydia, etc.). One recommended way to test for this is ANOSIM. Further exploration of the effects of different STI’s within STIpos is merited. Do any of the sub-groups cluster away from the STIneg? Do they exhibit differences with regards to skewing of immune cell subsets?

**Response 2:** Thank you for this comment. We did not see evidence that combining STI groups obscured findings for the individual pathogens, though we could certainly be underpowered to detect this. For each of the 23 blood and 26 rectal mucosal cell subsets, we conducted a one-way ANOVA test comparing the cell subset amounts for the GC, CT, TP, and multiple STI groups. Only one of these 49 tests – evaluating blood CD16-CD56+ NK cells - demonstrated a statistically significant (p < 0.05) difference between the groups (p = 0.02). We did not find any statistically significant difference between NK CD16-CD56+ cells in the blood related to STI or HIV status, and so it is possible that this could be because noise from the different STI subgroups obscured a correlation. It is also possible that this single p-value is positive due to multiple comparisons. We added more details to the limitations subsection of the Discussion section to address this reviewer’s concerns (bottom of pg. 20).

**Question 3.** The authors find that Chlamydia and Neisseria were enriched in the STIpos group consistent with the inclusion criteria for this group. The authors also find five other groups enriched in STIpos (Sneathia, Staphylococcus, Corynebacteriaceae, Bulleidia, and Hemophilus). Calling these taxa “pathogenic” is potentially problematic in that they are commensals that are present in healthy human skin (Staphylococcus, Corynebacteriaceae, and Hemophilus), vagina (Sneathia), and gut (Bulleidia). For example, Staphylococcus genus taxa are present in the skin of nearly every human sampled in large studies (<https://www.ncbi.nlm.nih.gov/pmc/articles/PMC4185404/>), and though Staphylococcus aureus is a well-known cause of harmful infection, most nares are colonized with it without overt infection or inflammation. “Ectopic” colonization of the gut with taxa from other body surfaces is associated with inflammation and these taxa can themselves stimulate inflammation (<https://pubmed.ncbi.nlm.nih.gov/29051379/>), so I believe the authors’ observations are of interest and are important, but calling them “pathogenic” may be an over-simplification. The authors may consider using the term of Atarashi et al. of ‘ectopic’ and noting these taxa typically colonize other body surfaces, or otherwise softening the idea that these are overt pathogens.

**Response 3:** Thank you for this thoughtful comment. We have replaced “pathogen” with “potential pathogen” throughout the microbiome sections of the manuscript. We reviewed the above references and did not feel that the term “ectopic’” was fully accurate in describing these taxa as some of these organisms have been shown to colonize the gut previously.

**Part III – Minor Issues: Editorial and Data Presentation Modifications**

**Reviewer #1:**

**Question 1.** The authors should include discussion of recent work in NHPs which suggest that the composition of the microbiome can influence SIV acquisition via low dose intrarectal challenge.

**Response 1:** Thank you for this comment. We have included discussion of these studies in the microbiome subsection of the Discussion section (top of pg.19).

**Question 2:** The authors should also discuss previous work (32415070) suggesting that the best predictor of HIV associated dysbiosis is nadir CD4 count, arguing that dysbiosis is not common in HIV until very late in the disease. The authors should also discuss previous work demonstrating that the microbiome of MSM is different than that of MSW (27077120).

**Response 2:** We have included discussion of these papers in the microbiome subsection of the Discussion section (bottom of pg. 19) as requested.

**Question 3.** The OTU bar graph in Figure 3 should be arranged by phyla.

**Response 3:** We replaced the amplicon sequence variant (ASV) bar graph in Figure 3 with two new bar graphs: one for the phyla as requested, and one for the genera (which is the depth at which our LDM analyses were conducted). We removed the original ASV bar graph, as this was conducted at the class level (our mistake). We also used a different program to make the current ASV graphs that allowed us to more accurately depict the most common taxa with unique colors for each.

**Question 4.** The use of antibiotics to treat the STIs is mentioned in the manuscript, but their use will obviously influence the composition of the microbiome. This needs to be further fleshed out in the figures and in the table describing the cohort. Which antibiotics, for how long, can the authors break up Abx treated individuals vs untreated in the figures? Do the authors have any longitudinal data (particularly on the microbiome) pre/post Abx use?

**Response 4:** As described in the Methods section (“The clinical cohort”), treatment of the bacterial STI was provided after the rectal mucosal and blood sampling procedures. Thus, antibiotic choice, duration, etc. did not impact any of the data collected for this study because antibiotics were provided after data collection. Unfortunately, we did not perform longitudinal sampling to assess the degree to which antibiotic treatment may have affected immune cell subsets, the transcriptome, or the microbiome. We agree that this is an exciting area of future research.

**Question 5:** The authors should provide representative flow cytometry gating strategies in the supplemental figures.

**Response 5:** We have added three supplementary figures (3, 4, and 5) with representative gating strategies.

**Reviewer #2:**

**Question 1:** Bacterial pathogens that spread by fecal:oral transmission like Campylobacter and Shigella have received attention for spreading sexually among MSM. Were these seen in this cohort?

**Response 1:** We are grateful to the reviewer for this insightful comment. Based on this and our prior oversight of including a class-level ASV graph in Figure 3A (as noted above), we visually re-examined our data at the genus level and specifically for *Campylobacter* and *Shigella*. To our surprise, these taxa were commonly detected in our cohort of YMSM (*Campylobacter* relative abundance 0.0092 and *Shigella* relative abundance 0.049). There were no differences in the relative abundance of *Campylobacter* or *Shigella* in the four study groups detected by LDM or Wilcoxon signed rank test which contributed to our initial oversight. Interestingly, we also noted that two specimens in our cohort appeared to be dominated by *Shigella*; both occurred in HIV–STI+ YMSM, one with GC and one with CT. Of note, we also examined the cellular subsets in these two participants and did not find them to be clear outliers with respect to the other specimens. This is a very interesting finding, and we will follow-up with additional, future analyses.

**Question 2:** Some minor typos grammatical:
a.Page 6 first line of results: “enrolled intro”
b.Page 10 last line: 16s should be 16S (same Page 11 second line and Fig 3 legend)

**Response 2:** Thank you for noticing these – we have made the corrections.

**Question 3:** Table 1: not all abbreviations defined in legend (e.g. GC, CT, TP)

**Response 3:** Thank you for noticing this – we added these definitions to the Table 1 legend.

**Question 4.** Would be nice to have more info on how “rectal biopsy and secretion sampling” was performed in main text– by “secretion sampling” are you referring to the rectal swabs for microbiome?

**Response 4:** We are referring to both the rectal swabs for microbiome analysis and pinch biopsies for immune cell subset and transcriptome analysis. We added a sentence explaining this to “The clinical cohort” subsection of the Results section (pg. 6).

**Question 5.** Please provide IRB approval number

**Response 5:** The IRB approval numbers are IRB00092959 and IRB000094847. We added this information to the clinical cohort subsection of the Methods section (bottom of pg. 22).

**Question 6.** Would be nice to have the gating strategies better described in this paper (supplemental materials if needed for space reasons) rather than just referencing previous publications. Same for the extraction and sequencing methods used to assess the microbiome.

**Response 6:** We have added three supplementary figures (3, 4, and 5) with representative gating strategies. We have added more details of the microbiome extraction and sequencing methods to the microbiota sequencing subsection of the Methods section (bottom of pg. 22).

**Question 7:** Page 9 line 2” confused by the statement “two-dimensional principal components analysis (PCAs) were constructed and analyzed by PERMANOVA” – I may be unfamiliar with this particular application but PERMANOVA is typically run on a distance matrix, not on the results of a PCA analysis. I have seen it coupled with principal coordinates analysis visualizations (PCoA) – because the PCoA might have been drawn using the same distance matrix. If this was done please indicate the distance metric used. If not, please explain in a little more detail here or in the methods how a “two-dimensional PCA” was performed and input into PERMANOVA. Also might want to consider using ADONIS because could evaluate HIV and STI in a single model.

**Response 7:** Thank you for noticing this discrepancy - you are correct. We used the top two PCs from PCoA to visualize our data. We then used a new version of PERMANOVA (PERMANOVA-FL, which was recently shown to outperform ADONIS – doi: 10.1093/bioinformatics/btaa260) embedded in the LDM package to analyze the association between a distance matrix and a trait of interest. We clarified our wording in this section of the manuscript (top of pg. 9) as well as in the immune cell subset statistical analyses subsection of the Methods section (bottom of pg. 26).

**Question 8:** Page 10 – for p-values reported – should give more detail on the statistical tests employed. E.g. for alpha diversity measures (Chao 1 etc.) – was this a T-test? For beta diversity (Bray-Curtis) was this PERMANOVA? For differential abundance – should consider using a compositionally aware analysis method like ANCOM-BC.

**Response 8:** Alpha diversity was tested by linear regression, since some covariates (such as the batch effect) need to be controlled for. PERMANOVA was used for beta-diversity. For differential abundance, we used LDM. As described in Response 7, we used a new version of PERMANOVA (PERMANOVA-FL, which was recently shown to outperform ADONIS – doi: 10.1093/bioinformatics/btaa260) embedded in the LDM package to analyze our data. LDM is not compositionally aware by design, but we elected to use it in order to avoid some inherent biases in compositionally aware methods, such as the biases associated with the introduction of pseudo counts and the incapability of handling rare taxa. It is proven to work well in cases when there are many taxa (such as 550 here) and the compositional effect is not very strong (Yang and Chen, Microbiome, 2022). Both approaches are accepted in the field. We updated the microbiome statistical analyses subsection of the Discussion section to include these details (pg. 26).

**Question 9:** Page 10: More details should be given somewhere on how microbiome beta diversity was calculated. Was this done on the ASV table or a taxa summary table and with what software package? Would suggest applying phylogenetically aware metric like unweighted/weighted UniFrac as could add more resolution potentially.

**Response 9:** Microbiome beta diversity was calculated using ASV table and with vegan software package. We added these details to the microbiome statistical analyses subsection of the Discussion section (pg. 27). There are many accepted distance measures for beta diversity, and Bray-Curtis is the most commonly used. We opted to use this over a phylogenetically aware method like UniFrac. To avoid false positives, we specified that we would consistently use Bray-Curtis for all beta diversity analysis from the outset rather than using multiple methods.

**Question 10:** Figure 3 B,C – and associated manuscript text on page 11 – should indicate statistical test used (PERMANOVA) should consider using UniFrac.

**Response 10:** We added this detail to the microbiome statistical analyses subsection of the Discussion section (pg. 26) and to the Figure 3 B, C legend. As described in Response 9, we used this over UniFrac throughout our analyses.

**Question 11:** Bottom page 11 – were these analyses done on genus level taxa and if so defined with which classifier? Methods list 2 different classifiers (Silva and GreenGenes) and things listed are something at genus and sometimes at family level.

**Response 11:** The analyses were done using the Silva classifier – we have edited the microbiota sequencing subsection of the Methods section to reflect this (bottom of pg. 22). The hits are not always at the same taxa level because the depth can sometimes vary. QIIME2 assigns the “deepest” taxonomy that it can find for a given 16s hit. If it is able to go to the genus or species level, it does, but some hits only make it to the phylum, family, etc. because either 1) the 16S gene at that level is identical, or 2) it is the extent to which sequence-able / culturable bacteria have been placed into the databases. Of the 11 taxa that were significantly different between the STI-positive and STI-negative groups, nine (*Neisseria, Chlamydia, Sneathia, Staphylococcus, Bulleidia, Hemophilus, Parabacteroides, Oscillibacter,* and *Phocea)* were classified to the genus level, and two (*Ruminococcaceae* and *Corynebacteriaceae*) were classified to the family level. We have added this information in every instance that these taxa are specifically mentioned in the manuscript.

**Question 12:** How were the 43 individuals selected for RNASeq analyses stratified across the 4 cohorts? (page 12)

**Response 12:** We restricted our analyses to those samples run in a single sequencing plate in order to avoid the risk of sequencing batch effect. The single batch we selected included the majority of participants and had representation from all four study groups included on the same plate. Other sequencing runs included only a few participants from a single study group, therefore we elected to exclude those specimens to avoid sequencing batch effects. We have added a statement to the RNA-seq statistical analyses subsection of the Methods section clarifying our selection of specimens for RNAseq analyses (top of pg. 27).

**Question 13:** Page 12 – it is stated that all of the significant genes that differed between YMSM with and without HIV contribute to immune function and tissue homeostasis. How was this determined and in what way?

**Response 13:** All of the 11 genes that were significant both in the global interaction analysis as well as the stratified analyses contribute to immune function and regulation or tissue homeostasis. We determined this by performing a literature review for each of these genes (listed below with brief summaries and example reference for each). We have also added the below citations to the text at the top of pg. 13. In the transcriptome section of the Discussion, we focused on a subset of these genes, as well as some of the genes that were highly significant in the stratified analyses only, in more detail in the interest of space.

B3GNT3 – Plays an important role in lymphocyte homing & trafficking. Downregulation of this gene enhances cytotoxic T-cell-mediated anti-tumor immunity (doi: 10.1016/j.ccell.2018.01.009).

AHNAK2 – Involved in innate immunity and the NF-KB pathway (doi: [10.1158/0008-5472.CAN-18-3845)](https://doi.org/10.1158/0008-5472.CAN-18-3845)). AHANAK2 methylation was also shown to contribute to HIV latency (doi: 10.1016/j.chom.2017.04.011)

TNIP3 – Also known as LIND/ABIN-3. An important negative regulator of innate immunity. It is induced by IL-10 and can block the NF-kB pathway from stimulation by IL-1, TLR, and TNF (doi: [10.1074/jbc.M607481200](https://doi.org/10.1074/jbc.M607481200)).

DMBT1 – Involved in immune defense and epithelial differentiation; its mRNA is expressed throughout the immune system, and some of its isoforms are identical to another protein with a known function in mucosal immune defense (PMID: 10749143)

TMEM47 – Plays an important role in the morphology and assembly rate of tight junctions (doi: [10.1002/dvdy.24404)](https://doi.org/10.1002%2Fdvdy.24404)

COL28A1- forms a collagen that associates with basement membranes (doi: [10.15252/msb.20156123)](https://doi.org/10.15252/msb.20156123))

HSPB7 – Modulates actin filament assembly, an important part of cell division (doi: 10.1073/pnas.1713763114)

ZNF132 – Involved in cell growth, migration, and invasion (doi: 10.1186/s12885-021-08112-z)

C4BPA – Mentioned in the manuscript discussion

C4BPB – Mentioned in the manuscript discussion

CASP5 – Mentioned in the manuscript discussion

**Question 14:** Last line of page 14 – I thought most prior studies show decrease of MAITs with HIV but you show increase, but says consistent with prior studies

**Response 14:** Homing of MAIT cells to sites of inflammation and microbial translocation, including the rectal mucosa, is a primary candidate explanation for the systemic loss of MAIT cells (described in detail in doi: https://doi.org/10.1038/s41467-019-13975-9). Our finding of increased MAIT cells in the gut mucosa is also consistent with systemic loss of MAIT cells as they home to the gut in PWH. Although we did not see a statistically significant difference in MAIT cells in the peripheral blood of our HIV+ and HIV– participants, we did see lower percentage of peripheral blood MAIT cells in our HIV+ YMSM that did not reach statistical significance (our data plotted below), which is again consistent with prior studies.

**Reviewer #3:**

**Question 1:** Abbreviations for the STIs will be helpful for readers if defined in the Table legend (Table 1) as they are in the text.

**Response 1:** Thank you – we added STI definitions to Table 1.
